# Benign Lipomatous Soft Tissue Tumors in Nigerians: An Analysis of the Clinical and Morphological Features of a Common but Important Entity

**DOI:** 10.7759/cureus.56618

**Published:** 2024-03-21

**Authors:** Kevin N Ezike, Ijeoma A Okwudire-Ejeh, Sule I Ahmed, Iliya K Salu, Michael E Aghahowa, Bamnan Dallang, Umar M Umar

**Affiliations:** 1 Anatomic Pathology and Forensic Medicine, Nile University of Nigeria, Abuja, NGA; 2 Anatomic Pathology and Forensic Medicine, Asokoro District Hospital, Abuja, NGA; 3 Orthopaedic Surgery, Asokoro District Hospital, Abuja, NGA; 4 Surgery, Nile University of Nigeria, Abuja, NGA; 5 Surgery, Asokoro District Hospital, Abuja, NGA; 6 Radiology, Nile University of Nigeria, Abuja, NGA

**Keywords:** lipoma variants, lipoma, nigeria, benign lipomatous, soft tissue

## Abstract

Introduction

Benign lipomatous tumors are soft tissue tumors that exhibit a predominant adipocytic phenotype. Lipomas are the archetype and are the most common benign soft tissue tumors in adults but relatively uncommon in children. Their sex incidence is equivocal. They sometimes occur in combination with other mesenchymal elements, giving rise to variants including fibrolipoma, angiolipoma, myolipoma, spindle cell lipoma, chondroid lipoma, osteolipoma, and chondrolipoma. Their clinical significance is mainly due to the cosmetic disfigurement of patients and the differential diagnosis of malignant soft tissue tumors. Occasionally, due to their large size or presence near vital organs, they may cause life-threatening and/or excruciating pressure symptoms.

This study was purposed to address the dearth of local studies on the clinical and morphological characteristics of benign lipomatous tumors in Nigerians, to compare these with those of other populations, and to establish baseline data.

Materials and methods

This was a retrospective study of all benign lipomatous tumors seen in the anatomic pathology and forensic medicine department of Asokoro District Hospital, Abuja, Federal Capital Territory, Nigeria, over an eight-year period. Surgical pathology reports were retrieved for patients’ biodata and clinical information. The appropriate slides were retrieved, and reviewed, and new sections were cut where necessary. The tumors were classified according to the 2020 World Health Organization (WHO) guidelines and categorized based on size as small, medium, or giant. The data obtained were analyzed, and the results were presented as tables, bar charts, ratios, and percentages.

Results

Four hundred and eighteen cases met the inclusion criteria. Of these, 58.4% (244/418), occurred in females, while 41.6% (174/418) occurred in males. The age range was six to 91 years, while the median age was 42 years. The least number of cases, 0.5% (2/418), were seen in patients aged less than 10 years, while the majority, 35.4% (148/418), occurred in the fifth decade, followed by 27.8% (116/418) in the fourth. Size-wise, the majority of tumors, 60% (253/418), were medium, followed by small, 22.8% (95/418). Giant-sized tumors significantly accounted for 16.7% (70/418) of the cases. The diagnostic spectrum comprised conventional lipoma and variants such as fibrolipoma, spindle cell lipoma, pleomorphic lipoma, angiolipoma, chondrolipoma, intramuscular lipoma, and osteolipoma. Lipoma and fibrolipoma dominated with 87.1% (364/418) and 10.0% (42/418), respectively, while the rest accounted for <3%. The majority, 31.8% (133/418), occurred in the back/shoulder region, followed by the lower limb with 18.2% (76/418). Only two cases occurred in the abdominal/pelvic region. More tumors occurred in females in all the regions except the head and neck, which had a male-to-female ratio of 1.5:1. Multiple site tumors were more common in males in a ratio of 2.5:1. Most, 41.1% (39/95), of the small-sized tumors, occurred in the head/neck region, largely involving the face, 48.7% (19/39).

Conclusion

Our study showed many similarities in the clinical and morphological features of benign lipomatous tumors between Nigerians and other regions of the world. A notable finding, however, was the significantly higher proportion of giant benign lipomatous tumors when compared to studies from other regions, a finding that warrants further studies.

## Introduction

Soft tissue is defined as the supportive tissue of various organs and the non-epithelial, extra-skeletal structures exclusive to lymphohematopoietic tissues [1]. It includes fibrous connective tissue, adipose tissue, skeletal muscle, blood/lymph vessels, and, by convention, the peripheral nervous system because tumors arising from nerves clinically resemble soft tissue masses and pose similar problems in differential diagnosis and therapy [1]. Embryologically, soft tissue is derived principally from the mesoderm, while peripheral nerves arise from the neuroectoderm [1]. Both benign and malignant tumors arise from soft tissue and are typically classified more according to the adult tissue they resemble than their histogenesis since the concept of histogenesis is not sustainable in some of these tumors [2].

Lipomatous tumors are soft tissue tumors with a predominant adipocytic component, and lipomas are the benign form [3]. Lipomas consist of adipocytes arranged in lobules and separated by septa of fibrous connective tissue [1]. They are occasionally associated with other mesenchymal elements, giving rise to different histopathological variants including fibrolipoma, angiolipoma, myolipoma, spindle cell lipoma, chondroid lipoma, osteolipoma, and chondrolipoma [3-5]. Lipomas are the most common soft tissue tumors, occur anywhere on the body, and are usually asymptomatic and superficial [6, 7]. They usually occur in adults above 30 years, peaking between ages 40 and 60 years, with variable statistics as to incidence in males and females [1]. They are, however, uncommon in children [1]. Being mostly asymptomatic, lipomas in subcutaneous locations are clinically significant because of the cosmetic discomfort and distress patients experience [8]. Lipomas in deeper locations, on the other hand, present with symptoms ranging from slight discomfort in joints and intramuscular locations to life-threatening respiratory distress related to bronchial obstruction when in endobronchial or parenchymal locations [8]. The major treatment for lipoma is surgical excision. However, in cases where malignancy is suspected, an initial incisional biopsy is done to establish a histological diagnosis.

The literature on the demographics, clinical features (site and size), and morphological characteristics of lipomas in Nigeria is very scanty, as so far only one study has been found in the literature [9]. Rather, a plethora of authors have reported on unusual sites and/or variants of lipomas [10-13]. This study attempts to fill that void by describing the clinical and morphological features of lipomas as seen in a district hospital in Nigeria to compare them with those of other populations and establish baseline data.

## Materials and methods

Study location and design

This was a retrospective study of all benign lipomatous tumors seen in the anatomic pathology and forensic medicine department of Asokoro District Hospital, Abuja, Federal Capital Territory (FCT), Nigeria, over an eight-year period from January 1, 2015, to December 31, 2022. Asokoro District Hospital is a quasi-tertiary center that serves as a referral center for cases from primary and secondary healthcare facilities in the FCT and other neighboring states such as Kogi, Nasarawa, and Niger States. It also provides teaching hospital services to the College of Health Sciences of Nile University of Nigeria, Abuja, FCT, Nigeria. Surgical pathology reports of patients diagnosed with benign lipomatous tumors during the study period were retrieved and scrutinized for macroscopic features. Patients’ biodata and clinical information were retrieved from electronic archives. The appropriate slides were retrieved, and reviewed, and new sections were cut from formalin-fixed, paraffin-embedded blocks in cases where such slides were missing or faded. All biopsies had been fixed in 10% neutral buffered formalin. The retrieved and freshly cut slides were reviewed by three pathologists with concurrence in equivocal cases in order to minimize interobserver variations.

Inclusion and exclusion criteria

Inclusion Criteria

Cases where the patient’s complete biodata and clinical information, along with a pathology report of lipoma/benign lipomatous tumors, were available and the slides were successfully reviewed were selected for the study. Also selected were cases that met the above criteria but for which only information on the tumor site was available.

Exclusion Criteria

Cases with the complete absence of either the patient’s biodata or clinical information and cases with missing slides for which the paraffin-embedded tissue blocks could not be found were excluded from the study.

Diagnostic criteria

The tumors were classified according to the 2020 World Health Organization (WHO) Classification of Soft Tissue Tumors [14]. The tumors were categorized based on size as small, medium, or giant, according to the definitions of Charifa et al. and Serpell et al. [15,16]. The specific sites of the tumors were grouped for convenience into regions as follows: head/neck, anterior trunk, back/shoulder, arm/axilla, pelvis/perineum, lower limb, and abdominal/pelvic cavities. Those that occurred at multiple sites were grouped, and those that met the inclusion criteria but for whom the location was not specified were categorized as not specified.

Statistical analysis

All data were entered in a spreadsheet using the Microsoft Office 365 version of the Excel software program (Microsoft Corporation, Redmond, WA) and analyzed with IBM SPSS Statistics for Windows, version 24.0 (IBM Corporation, Armonk, NY). Continuous variables were summarized using descriptive statistics, including range, median, mean ± standard deviation, and confidence interval, and analyzed using the chi-square test statistic. A p-value <0.05 was considered statistically significant. The results were displayed using tables and charts.

Ethical consideration

Ethical clearance was obtained from the medical ethics committee of Asokoro District Hospital (approval number: FCT/HHSS/HMB/ADH/139/23.)

## Results

A total of 418 cases of benign lipomatous tumors diagnosed within the study period met the inclusion criteria. Of these, 58.4% (244/418) occurred in females, while 41.6% (174/418) occurred in males, giving a male-to-female ratio of 1:1.4.

**Figure 1:**
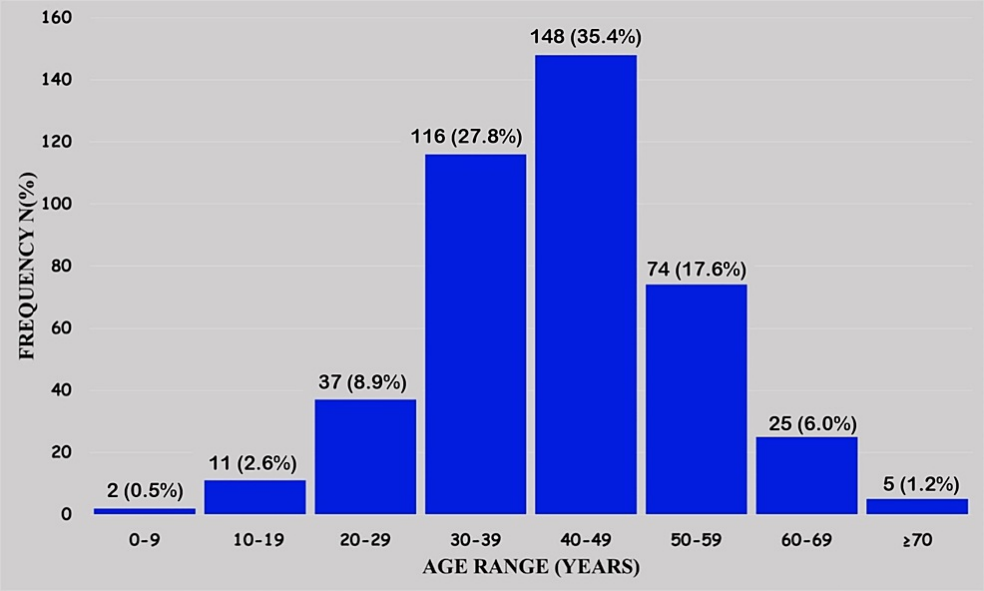
Cases of benign lipomatous tumors distributed according to age

The mean (mean±SD) of the patients’ ages was 41.9±11.6 years, with a range of six to 91 years, while the median age was 42 years. The confidence interval was 40.9-43.1. The least number of cases were seen in patients aged less than 10 years, who accounted for just 0.5% (2/418), and these occurred on the neck and back, respectively, of a male aged six years and a female aged eight years. The majority of the cases occurred in the 40-49 year age range with 35.4% (148/418), followed by the 30-39 year age range with 27.8% (116/418). Indeed, both age ranges combined accounted for 63.2% (264/418) of cases (Figure 1)

**Figure 2:**
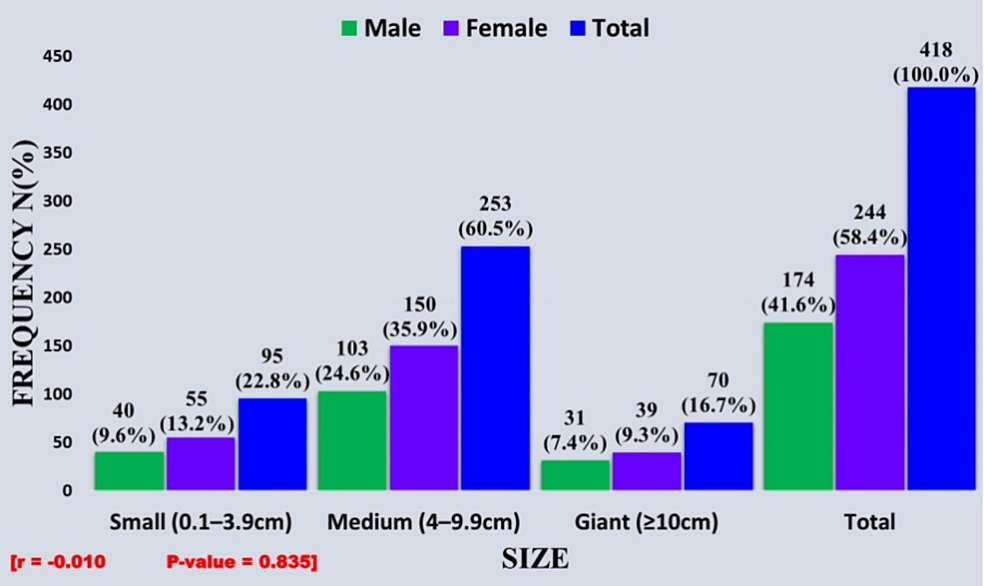
Distribution of benign lipomatous tumors as per size and sex

In terms of size, the largest tumor was a fibrolipoma, which measured 24 x 16 x 12cm and was located in the right thigh of a 45-year-old male, while the smallest was a lipoma, which measured 0.6 x 0.5 x 0.2cm and was located in the left popliteal fossa of a 50-year-old male. The mean size of the tumors was 6.4±3.6cm with a range of 1 cm to 24 cm, while the median size was 6 cm. The confidence interval was 6.1-6.8.1. Medium-sized tumors (4 cm-9.9 cm) accounted for 60% (253/418) of cases and were therefore in the majority. The relationship between the size of the tumor and the sex of the patients was not statistically significant (Figure 2).

In the various size groups, the majority of the tumors were seen in the 40-49 year age range as follows: small, 38.9% (37/95); medium, 31.2% (79/253); and giant, 45.7% (32/70). Thus, the relationship between the age of patients and the size of the tumor was not statistically significant as it followed the overall age distribution. However, the correlation coefficient (r = -0.0019) indicates a weak negative association between age and tumor size (Table 1).

**Table 1:** Distribution of benign lipomatous tumors as per age and size

Age range (years)	Size		
	Small (0.1 cm–3.9 cm) n(%)	Medium (4 cm–9.9 cm) n(%)	Giant (≥10cm) n(%)
0–9	0 (0.0)	2 (0.8)	0 (0.0)
10–19	4 (4.2)	5 (2.0)	2 (2.9)
20–29	7 (7.4)	26 (10.3)	4 (5.7)
30–39	22(23.2)	78(30.8)	16(22.8)
40–49	37(38.9)	79(31.2)	32(45.7)
50–59	18 (18.9)	45 (17.8)	11 (15.7)
60–69	7 (7.4)	15 (5.9)	3 (4.3)
≥70	0 (0.0)	3 (1.2)	2 (2.9)
Total	95 (100.0)	253 (100.0)	70 (100.0)
Correlation coefficient (r) = –0.0019			
P–value = 0.990			

**Figure 3:**
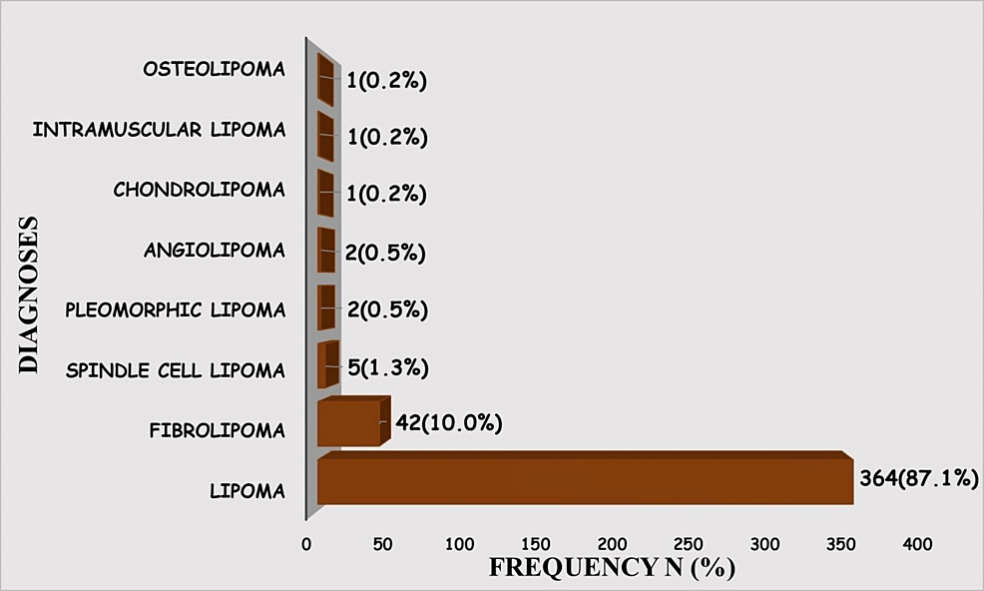
Diagnostic spectrum of benign lipomatous tumors

The diagnostic spectrum of tumors was varied and comprised a significant representation of conventional lipoma and lipoma variants, including fibrolipoma, spindle cell lipoma, pleomorphic lipoma, angiolipoma, chondrolipoma, intramuscular lipoma, and osteolipoma. Lipoma and fibrolipoma dominated with 87.1% (364/418) and 10.0% (42/418), respectively. The other diagnoses combined were less than 3% (Figure 3).

The relationship between diagnoses and the size of these benign lipomatous tumors was not statistically significant. In keeping with the overall size and number distribution, both lipoma and fibrolipoma, which were the most frequent diagnoses, occurred more in the medium size category, at 62.4% (227/364) and 54.8% (23/42), respectively (Table 2).

**Table 2:** Distribution of benign lipomatous tumors according to the diagnosis and size

Diagnoses	Size			Total n(%)
	Small n(%)	Medium n(%)	Giant n(%)	
Lipoma	77 (21.2)	227 (62.4)	60 (16.4)	364 (100)
Fibrolipoma	11 (26.2)	23 (54.8)	8 (19.0)	42 (100)
Spindle cell lipoma	3 (60.0)	1 (20.0)	1 (20.0)	5 (100)
Angiolipoma	1 (50.0)	0 (0.0)	1 (50.0)	2 (100)
Pleomorphic lipoma	1 (50.0)	1 (50.0)	0 (0.0)	2 (100)
Chondrolipoma	1 (100.0)	0 (0.0)	0 (0.0)	1 (100)
Intramuscular lipoma	0 (0.0)	1 (100.0)	0 (0.0)	1 (100)
Osteolipoma	0 (0.0)	1 (100.0)	0 (0.0)	1 (100)
Chi-square = 17.308				
P–value = 0.696				

**Figure 4:**
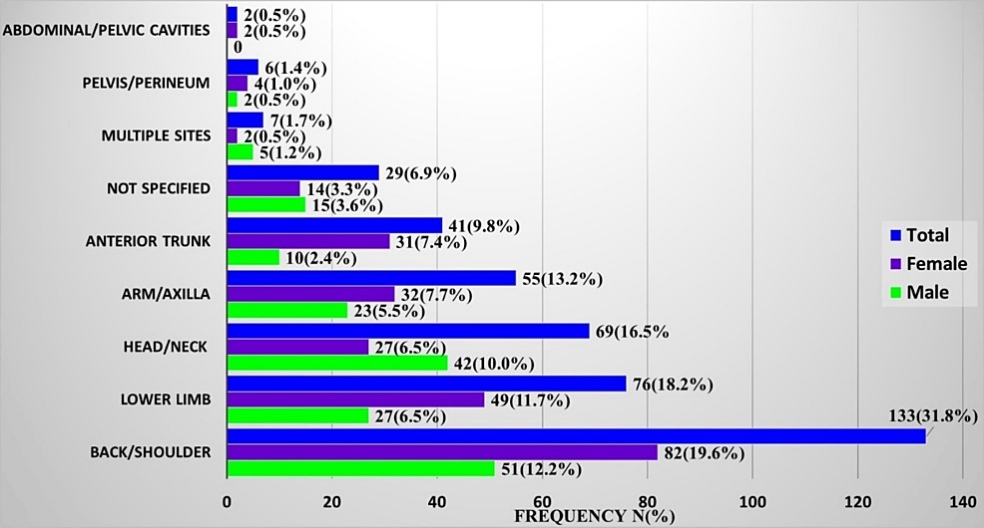
Distribution of benign lipomatous tumors according to the region of occurrence and sex

The relationship between the region or site and the sex distribution of these tumors was statistically significant. Overall, the commonest region of occurrence was the back/shoulder region with 31.8% (133/418) of cases, followed by the lower limb region with 18.2% (76/418). In keeping with the general sex distribution, more tumors occurred in females in all the regions except the head and neck, which had more tumors in males in a ratio of 1.5:1. Cases in which tumors occurred at multiple sites also showed a male preponderance with a ratio of 2.5:1 (Figure 4).

The highest number of tumors occurred in the back and shoulders, both accounting for 21.1% (88/418), with a male-to-female ratio of 1:1.5. The least number, 0.5% (2/418), occurred in the abdominal/pelvic cavity in two females. In the lower limb region, the majority of the tumors, 64.5% (49/76), occurred in the thigh with a male-to-female ratio of 1:2. In the anterior trunk region, the chest with 46.3% (19/41) was the most frequent site of occurrence, with a male-to-female ratio of 1:3. However, in the breast, of the 12 cases seen, 11 occurred in females (Table 3).

**Table 3:** Distribution of benign lipomatous tumors according to region/site of occurrence and sex

Site	Frequency		Total n(%)
	Male n(%)	Female n(%)	
Head/Neck region			
Face	15 (21.7)	8 (11.6)	23 (33.3)
Neck	11 (15.9)	13 (18.8)	24 (34.8)
Scalp	12 (17.4)	2 (2.9)	14 (20.3)
Eye	2 (2.9)	2 (2.9)	4 (5.8)
Ear	1 (1.4)	1 (1.4)	2 (2.9)
Lower lip	1 (1.4)	1 (1.4)	2 (2.9)
Total n(%)	42 (60.9)	27 (39.1)	69 (100)
Back/Shoulder region			
Back	34 (25.6)	44 (33.1)	78 (58.6)
Shoulder	16 (12.0)	29 (21.8)	45 (33.8)
Scapular	1 (0.8)	9 (6.8)	10 (7.5)
	51 (38.3)	82 (61.7)	133 (100.0)
Arm/Axilla region			
Arm	19 (4.5)	24 (5.7)	43 (10.3)
Axilla	4 (1.0)	8 (1.9)	12 (2.9)
Total	23 (5.5)	32 (7.7)	55 (13.2)
Anterior trunk region			
Chest	6 (14.6)	13 (31.7)	19 (46.3)
Anterior abdominal wall	3 (7.3)	7 (17.1)	10 (24.4)
Breast	1 (2.4)	11 (26.8)	12 (29.3)
Total	10 (24.4)	31 (75.6)	41 (100.0)
Lower limb region			
Gluteus	8 (10.5)	10 (13.2)	18 (23.7)
Leg	3 (3.9)	6 (7.9)	9 (11.8)
Thigh	16 (21.1)	33 (43.4)	49 (64.5)
Total	27 (35.5)	49 (64.5)	76 (100.0)
Pelvis/Perineum			
Perineum	2 (33.3)	2 (33.3)	4 (66.7)
Vulva	0 (0.0)	2 (33.3)	2 (33.3)
Total	2 (33.3)	4 (66.7)	6 (100.0)
Abdominal/Pelvic cavities			
Omentum	0 (0.0)	1 (50.0)	1 (0.2)
Ovary	0 (0.0)	1 (50.0)	1 (0.2)
Total	0 (0.0)	2 (100.0)	2 (100.0)
Multiple sites			
Multiple sites	5 (71.4)	2 (28.6)	7 (100.0)
Not specified			
Not specified	15 (51.7)	14 (48.3)	29 (100.0)
Chi-square = 22.195			
P–value = 0.005 (significant at 95%)			

The association between the region/site and the size of the tumors was statistically significant. More of the small tumors, 41.1% (39/95), were seen in the head/neck region, with the face, 20.0% (19/95), accounting for the majority of these. Unusual locations in the face included the conjunctiva, eyelid, and lower lip. The next most common region of small tumor location was the lower limb, which had 17.9% (17/95), and the thigh, 14.7% (14/95), had the highest number in this region. The medium-sized tumors were commonest in the back/shoulder region with 34.9 (88/253) while significant numbers occurred in the arm/axilla, 16.6% (42/253), and lower limb, 16.2% (41/253), regions. Out of the 30 medium-sized tumors seen in the head/neck region, 60% (18/30) occurred in the neck. The back/shoulder region, with 44.3% (31/70), was the most frequent location of giant-sized tumors, followed by the lower limb region, which had 25.7% (18/70), with the majority of these (14/18) occurring in the thigh (Table 4). 

**Table 4:** Distribution of benign lipomatous tumors as per region/site of occurrence and size

Region	Site	Size					
		Small n(%)	Total n(%)	Medium n(%)	Total n(%)	Giant n(%)	Total n(%)
Head/ Neck	Face	19 (4.5)	39 (9.3)	4 (1.0)	30 (7.2)	0 (0.0)	0 (0.0)
	Neck	5 (1.2)		18 (4.3)		0 (0.0)	
	Scalp	8 (1.9)		7 (1.7)		0 (0.0)	
	Eye	4 (1.0)		0 (0.0)		0 (0.0)	
	Ear	1 (0.2)		1 (0.2)		0 (0.0)	
	Lower Lip	2 (0.5)		0 (0.0)		0 (0.0)	
Back/Shoulder	Back	8 (1.9)	14 (3.3)	46 (11.0)	88 (21.1)	24 (5.7)	31 (7.4)
	Shoulder	4 (1.0)		37 (8.9)		4 (1.0)	
	Scapular	2 (0.5)		5 (1.2)		3 (0.7)	
Perineum/ Pelvis	Perineum	2 (0.5)	2 (0.5)	2 (0.5)	3 (0.7)	0 (0.0)	1 (0.2)
	Vulva	0 (0.0)		1 (0.2)		1 (0.2)	
Lower Limb	Gluteus	2 (0.5)	17 (4.1)	14 (3.3)	41 (9.8)	2 (0.5)	18 (4.3)
	Leg	1 (0.2)		6 (1.4)		2 (0.5)	
	Thigh	14 (3.3)		21 (5.0)		14 (3.3)	
Anterior trunk	Chest	1 (0.2)	7 (1.7)	17 (4.1)	28 (6.7)	1 (0.2)	6 (1.4)
	Anterior abdominal wall	2 (0.5)		4 (1.0)		4 (1.0)	
	Breast	4 (1.0)		7 (1.7)		1 (0.2)	
Arm/Axilla	Arm	6 (1.4)	7 (1.7)	33 (7.9)	42 (10.0)	4 (1.0)	6 (1.4)
	Axilla	1 (0.2)		9 (2.2)		2 (0.5)	
Abdominal/ Pelvic cavities	Omentum	1 (0.2)	2 (0.5)	0 (0.0)	0 (0.0)	0 (0.0)	0 (0.0)
	Ovary	1 (0.2)		0 (0.0)		0 (0.0)	
Multiple sites	Multiple sites	1 (0.2)	1 (0.2)	4 (1.0)	4 (1.0)	2 (0.5)	2 (0.5)
Not specified	Not specified	6 (1.4)	6 (1.4)	17 (4.1)	17 (4.1)	6 (1.4)	6 (1.4)
Total		95 (22.7)		253 (60.5)		70 (16.7)	
Chi-square = 77.035							
P–value = 0.001 (significant at 95%)							

**Figure 5:**
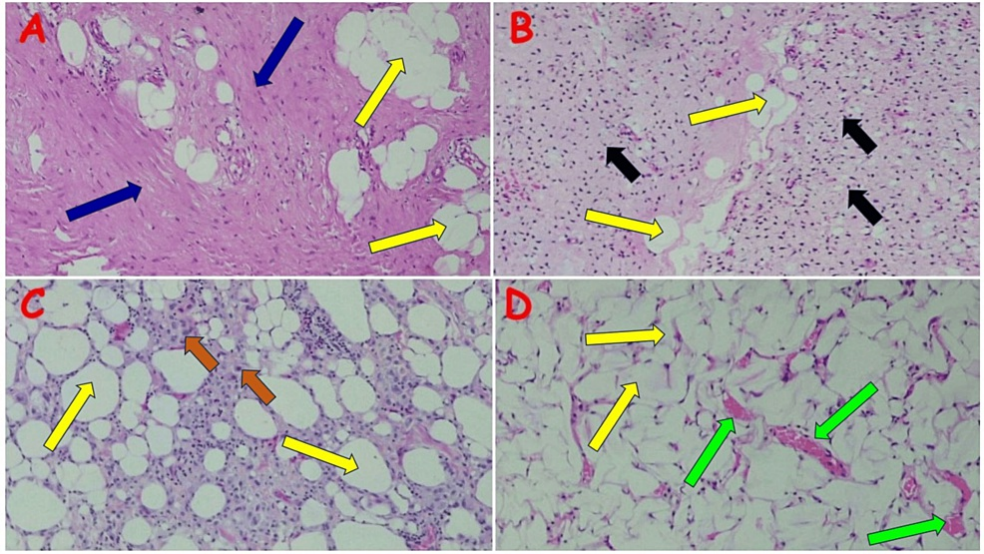
Fibrolipoma and other lipoma variants (A) Giant fibrolipoma in the right thigh of a 45-year-old male; note the lobules of mature adipocytes (yellow arrows) traversed by vascularized fibrous tissue bands (blue arrows). (B) Spindle cell lipoma in the right thigh of a 35-year-old female; note the admixture of mature adipocytes (yellow arrows) and spindle-shaped cells (black arrows) set in the fibromyxoid stroma. (C) Pleomorphic lipoma on the scalp of a 36-year-old male; note the variably sized adipocytes (yellow arrows) set in the hyalinized spindle cell stroma, admixed with numerous floret cells (brown arrows). (D) Angiolipoma in the right arm of a 19-year-old female; note the admixture of mature adipocytes (yellow arrows) and often ectatic capillary-type blood vessels, which occasionally contain bright pink fibrin (green arrows).

The relationship between the region/site distribution and the specific diagnoses of the tumors was statistically significant. In keeping with the overall site distribution, the most common tumors, which were lipomas, were more common in the back/shoulder region with 34.8% (127/364), while fibrolipomas occurred more frequently in the lower limb with 28.6% (12/42). Three other tumors occurred two times or more, namely spindle cell lipoma, which occurred five times (twice each in the neck and thigh and once in the back); pleomorphic lipoma, which occurred once each in the scalp and right thigh; and angiolipoma, which occurred once each in the perineum and right arm (Table 5 and Figure 5).

**Table 5:** Distribution of benign lipomatous tumors according to the region/site of occurrence and diagnosis

Region	Site	Diagnoses							
		Chondrolipoma	Fibrolipoma	Spindle cell lipoma	Lipoma	Pleomorphic lipoma	Angiolipoma	Osteolipoma	Intramuscular lipoma
Head and neck	Face	0 (0.0)	4 (9.5)	0 (0.0)	18 (4.9)	0 (0.0)	0 (0.0)	0 (0.0)	0 (0.0)
	Neck	0 (0.0)	3 (7.1)	2 (40.0)	19 (5.2)	0 (0.0)	0 (0.0)	0 (0.0)	0 (0.0)
	Scalp	0 (0.0)	2 (4.8)	0 (0.0)	12 (3.3)	1 (50.0)	0 (0.0)	0 (0.0)	0 (0.0)
	Eye	0 (0.0)	0 (0.0)	0 (0.0)	4(1.1)	0 (0.0)	0 (0.0)	0 (0.0)	0 (0.0)
	Ear	0 (0.0)	1 (2.4)	0 (0.0)	1 (0.3)	0 (0.0)	0 (0.0)	0 (0.0)	0 (0.0)
	Lower lip	1 (100.0)	0 (0.0)	0 (0.0)	1 (0.3)	0 (0.0)	0 (0.0)	0 (0.0)	0 (0.0)
Back/Shoulder	Back	0 (0.0)	2 (4.8)	1 (20.0)	75 (20.6)	0 (0.0)	0 (0.0)	0 (0.0)	0 (0.0)
	Shoulder	0 (0.0)	3 (7.1)	0 (0.0)	42 (11.5)	0 (0.0)	0 (0.0)	0 (0.0)	0 (0.0)
	Scapular	0 (0.0)	0 (0.0)	0 (0.0)	10 (2.7)	0 (0.0)	0 (0.0)	0 (0.0)	0 (0.0)
Perineum/Pelvis	Perineum	0 (0.0)	0 (0.0)	0 (0.0)	3 (0.8)	0 (0.0)	1 (50.0)	0 (0.0)	0 (0.0)
	Vulva	0 (0.0)	1 (2.4)	0 (0.0)	1 (0.3)	0 (0.0)	0 (0.0)	0 (0.0)	0 (0.0)
Lower limb	Gluteus	0 (0.0)	2 (4.8)	0 (0.0)	16 (4.4)	0 (0.0)	0 (0.0)	0 (0.0)	0 (0.0)
	Leg	0 (0.0)	0 (0.0)	0 (0.0)	8 (2.2)	0 (0.0)	0 (0.0)	1 (100.0)	0 (0.0)
	Thigh	0 (0.0)	10 (23.8)	2 (40.0)	35 (9.6)	1 (50.0)	0 (0.0)	0 (0.0)	1 (100.0)
Anterior trunk	Chest	0 (0.0)	0 (0.0)	0 (0.0)	19 (5.2)	0 (0.0)	0 (0.0)	0 (0.0)	0 (0.0)
	Anterior abdominal wall	0 (0.0)	1 (2.4)	0 (0.0)	9 (2.5)	0 (0.0)	0 (0.0)	0 (0.0)	0 (0.0)
	Breast	0 (0.0)	0 (0.0)	0 (0.0)	12 (3.3)	0 (0.0)	0 (0.0)	0 (0.0)	0 (0.0)
Arm/Axilla	Arm	0 (0.0)	5 (11.9)	0 (0.0)	37 (10.2)	0 (0.0)	1 (50.0)	0 (0.0)	0 (0.0)
	Axilla	0 (0.0)	1 (2.4)	0 (0.0)	11 (3.0)	0 (0.0)	0 (0.0)	0 (0.0)	0 (0.0)
Abdominal/Pelvic cavities	Omentum	0 (0.0)	0 (0.0)	0 (0.0)	1 (0.3)	0 (0.0)	0 (0.0)	0 (0.0)	0 (0.0)
	Ovary	0 (0.0)	0 (0.0)	0 (0.0)	1 (0.3)	0 (0.0)	0 (0.0)	0 (0.0)	0 (0.0)
Multiple sites	Multiple sites	0 (0.0)	2 (4.8)	0 (0.0)	5 (1.4)	0 (0.0)	0 (0.0)	0 (0.0)	0 (0.0)
Not specified	Not specified	0 (0.0)	5 (11.9)	0 (0.0)	24 (6.6)	0 (0.0)	0 (0.0)	0 (0.0)	0 (0.0)
Total n(%)		1 (100.0)	42 (100.0)	5 (100.0)	364 (100)	2 (100.0)	2 (100.0)	1 (100.0)	1 (100.0)
Chi-square = 79.039									
P–value = 0.023 (significant at 95%)									

**Figure 6:**
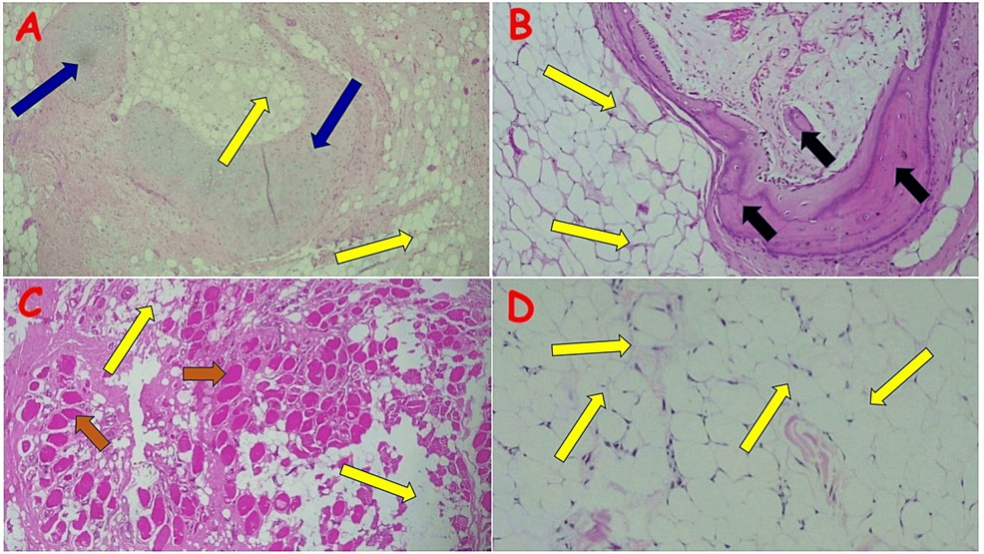
Other rare lipoma variants and conventional lipoma (A) Chondrolipoma in the lower lip of a 50-year-old male [10]; note the mature adipose tissue (yellow arrows) separated by faintly basophilic fibrous septae admixed with foci of metaplastic hyaline cartilage (blue arrows). (B) Osteolipoma in the right foot of a 19-year-old male; note the lobules of mature adipocytes (yellow arrows) admixed with and completely surrounding the trabeculae of mature bone (black arrows). (C) Intramuscular lipoma in the left thigh of a 37-year-old male; note the mature adipocytes (yellow arrows) interdigitating with skeletal muscle fibers (brown arrows). (D) Conventional lipoma on the right conjunctiva of a 40-year-old male; note the lobules of mature adipocytes (yellow arrows) demarcated into lobules by delicate fibrous strands. Image A: Reproduced with permission from Ezike KN, Okwudire-Ejeh IA, Essien LO, Chukwu FO [10]

The rare lipoma variants diagnosed tended to also occur in unusual locations. Chondrolipoma occurred in the lower lip of a 50-year-old male [10]. The only osteolipoma diagnosed was in the right foot of a 19-year-old male, while the intramuscular lipoma occurred in the left thigh of a 37-year-old male. The conventional lipoma also occurred at unusual sites, including the conjunctiva, ovary, lower lip, and right middle finger (Figure 6).

## Discussion

Benign lipomatous tumors are mesenchymal tumors, composed of mature, histologically benign adipocytes, occasionally admixed with other mesenchymal elements such as fibroblasts, blood vessels, chondroblasts, smooth muscle, skeletal muscles, and other heterologous elements [4,8]. They are, however, histogenetically distinct from entities like lipomatous mixed tumors and mature cystic teratoma, which are skin adnexal and germ cell tumors, respectively, but exhibit significant adipocytic differentiation [17,18]. The pathophysiology of benign lipomatous tumors is not fully understood. However, two theories, including the genetic and the trauma pathogenesis theories, are postulated. The most common genetic abnormality seen in these tumors is the rearrangement of chromosome 12, which contains the DNA damage-inducible transcript 3 (DDIT3) gene, whose protein family, enhancer binding protein, is involved in the growth arrest of fat cells [19]. Etiologically, an association has been made between trauma and lipoma formation with the action of growth factors and cytokines released in response implicated in its pathogenesis; however, this has not been unequivocally demonstrated [8]. Neither the demonstrated genetic abnormalities, including chromosome 12 rearrangements, nor the trauma pathogenesis theory have any significant association with the sex or age of patients who develop lipomas or the tumor site, size, or depth [20].

There is no consensus on the overall sex incidence of benign lipomatous tumors. While some authorities and researchers say it is more common in males, others report an equal incidence, and some report a female preponderance [8, 9,20,21]. Our study with a male-to-female ratio of 1:1.5 aligns with the female preponderance. Within different compartments, however, variations in sex incidence have been reported. For example, according to many studies, head and neck lipomas are more prevalent in males, and similar to these studies, our study showed a higher prevalence in males, with a ratio of 1.5:1 [22-25].

Benign lipomatous tumors occur in all age groups and demographics but are more common in persons aged 30-60 years [1,2,9,16,24-26]. Our study, with findings from 81% of cases within this age range, did not buck this trend. These tumors, however, seem to peak at an earlier age in Nigerians, in keeping with an earlier Nigerian study by Dauda et al. [9], unlike studies in Indian and Swedish populations that showed a peak incidence in the 40-60-year range, respectively [26,27].

Giant benign lipomatous tumors (greater than 10 cm in widest diameter) are rare, and the vast majority of benign lipomatous tumors are considered small to medium-sized, measuring less than 6 cm in widest diameter [1,2,27]. Our study showed a relatively high proportion of giant lipomas (16%) in contrast with the 0.01% and 0.07% reported by Rydholm et al. and Mello et al., respectively [27,28]. The reasons for this are unclear, however, our cases occurred almost exclusively in a Black African population, while the two studies referenced above were in Latin American and European populations, respectively. A racial or genetic predisposition to giant lipomas may, therefore, be a factor in our study population. It is also possible that patients in our study tended to ignore the swelling until it reached a larger size [29]. Further research is required to test this hypothesis.

The relationship between the size of these tumors and the sex of the patients is not statistically significant, and our findings corroborate the same [27]. In terms of size and site of the tumors, our series showed that the back and shoulders had larger tumors than the head and neck region, findings similar to other studies both within and outside Nigeria [9,27].

The differential diagnoses of benign lipomatous tumors depend on site and depth and range from sebaceous cysts and abscesses in superficial locations to liposarcoma, including atypical lipomatous tumors, dermatofibroma, dermatofibrosarcoma protuberance (DFSP), neurofibroma, etc., in subfascial and deep locations [1,8]. Conversely, benign lipomatous tumors should be considered in the differential diagnosis of all superficial masses and masses located in specific compartments of the body.

Limitations

The major limitation of the study was that it was hospital-based and therefore analyzed only the cases that underwent surgical excision. As a result, the findings may not reflect the true prevalence and incidence of benign lipomatous tumors within the population. Another limitation is the retrospective nature of the study design, which was associated with incomplete data, especially tumor location, with possible distortion of the location statistics.

## Conclusions

Our study demonstrates that the clinical and morphological features of benign lipomatous features in Nigerians are generally similar to findings reported from other regions of the world. A notable finding, however, was the significantly higher proportion of giant benign lipomatous tumors when compared to studies from these other regions. Multicenter studies are required to corroborate this finding and determine whether or not a racial or genetic predisposition to giant lipomas exists in our population. Furthermore, surgical excision of these tumors should be encouraged not only for cosmetic reasons and/or pressure symptoms but also because of the need to rule out malignant differential diagnoses.
